# Genotype data not consistent with clonal transmission of sea turtle fibropapillomatosis or goldfish schwannoma

**DOI:** 10.12688/wellcomeopenres.17073.1

**Published:** 2021-09-02

**Authors:** Máire Ní Leathlobhair, Kelsey Yetsko, Jessica A. Farrell, Carmelo Iaria, Gabriele Marino, David J. Duffy, Elizabeth P. Murchison

**Affiliations:** 1 Ludwig Institute for Cancer Research, Nuffield Department of Clinical Medicine, University of Oxford, UK; 2 Big Data Institute, University of Oxford, Oxford, UK; 3 Transmissible Cancer Group, Department of Veterinary Medicine, University of Cambridge, Cambridge, UK; 4 The Whitney Laboratory for Marine Bioscience, Sea Turtle Hospital, University of Florida, St. Augustine, Florida, 32080, USA; 5 Department of Biology, University of Florida, Gainesville, Florida, 32611, USA; 6 Centre of Experimental Fish Pathology of Sicily (CISS), Viale Giovanni Palatucci, University of Messina, 98168, Messina, Italy; 7 Department of Chemical, Biological, Pharmaceutical and Environmental Sciences, Viale Ferdinando Stagno d'Alcontres, n 31, University of Messina, 98166, Messina, Italy; 8 Department of Veterinary Sciences, Viale Giovanni Palatucci, University of Messina, 98168, Messina, Italy

**Keywords:** sea turtle fibropapillomatosis, fibropapillomatosis, goldfish schwannoma, transmissible cancer, wildlife cancer

## Abstract

Recent discoveries of transmissible cancers in multiple bivalve species suggest that direct transmission of cancer cells within species may be more common than previously thought, particularly in aquatic environments. Fibropapillomatosis occurs with high prevalence in green sea turtles ( *Chelonia mydas*) and the geographic range of disease has increased since fibropapillomatosis was first reported in this species. Widespread incidence of schwannomas, benign tumours of Schwann cell origin, reported in aquarium-bred goldfish *(Carassius auratus), *suggest an infectious aetiology. We investigated the hypothesis that cancers in these species arise by clonal transmission of cancer cells. Through analysis of polymorphic microsatellite alleles, we demonstrate concordance of host and tumour genotypes in diseased animals. These results imply that the tumours examined arose from independent oncogenic transformation of host tissue and were not clonally transmitted. Further, failure to experimentally transmit goldfish schwannoma via water exposure or inoculation suggest that this disease is unlikely to have an infectious aetiology.

## Introduction

Cancer is an increasingly recognised cause of mortality in many domestic and wildlife animal species ^1– 3^. Clusters of neoplastic disease cases can be linked to species-specific genetic vulnerabilities ^4^, environmental contaminant exposure ^5^ and infectious aetiologies ^6, 7^. However, in the latter case, the causative infectious agent often remains elusive ^1, 2^. One infectious modality that may be more frequent than previously assumed is the transmissible cancer cell ^7, 8^. Transmissible cancers are somatic cell lineages that are spread between hosts by the physical transfer of living cancer cells. These clones can ‘metastasize’ within populations, having adapted to transmit across external environments and evade host immune responses. Ten naturally occurring transmissible cancer lineages have been described: one in domestic dogs ^9– 11^, two lineages in Tasmanian devils ^12, 13^ as well as multiple independent lineages in marine bivalves ^14– 17^. In this study, we assessed the hypothesis of clonal transmission in two animal cancers.

**Figure 1.**
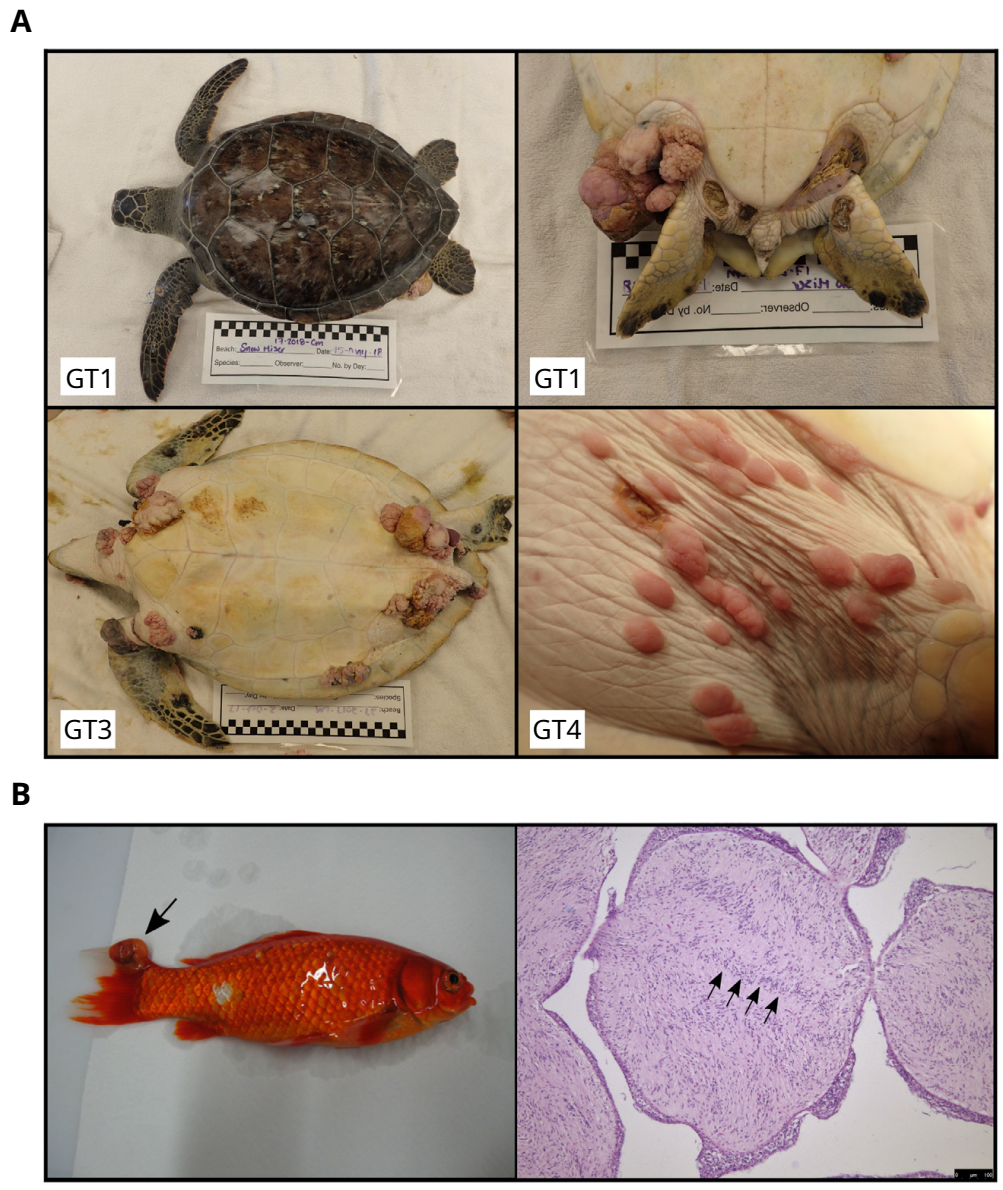
Gross appearance of sea turtle fibropapillomatosis and goldfish schwannoma. ( **A**) Gross appearance of sea turtle fibropapillomatosis (FP) in *C. mydas* individuals collected for this study at the Whitney Sea Turtle Hospital, University of Florida. Upper panel, Patient 1 (GT1): dorsal image (left) and established tumour on left rear flipper (right). Lower panel, Patient 3 (GT3) showing multiple established lesions around ventral tail, inguinal regions, plastron, axillary regions, neck and front flippers (left), and Patient 4 (GT4) showing new-growth tumours on the inguinal regions (right). ( **B**) Left, gross appearance of goldfish schwannoma in an individual collected for this study. Tail fin tumour indicated with an arrow. Right, representative tumour histology from a haematoxylin and eosin stained section. Nuclear palisading, which is diagnostic for goldfish schwannoma can be observed (arrows) (right).

Fibropapillomatosis (FP) is a neoplastic disease reported in all seven sea turtle species ^18– 22^. FP results in fibroepithelial lesions that are often associated with the external soft tissues, with common sites including the flippers, inguinal and axillary regions, oral cavity and conjunctiva ( Figure 1, Table 1). Tumours affecting the visceral organs, such as lungs, kidneys, heart, and liver, are also reported. Although usually localised, secondary complications arising from tumour site and tumour burden can limit host lifespan by impairing vision, feeding, and internal organ function. The first report of FP was made in 1938, when disease was described in a captive green sea turtle ( *Chelonia mydas*) from Key West, Florida ^23^. The disease is now recognised in *C. mydas* populations worldwide and could threaten long-term population survival given higher disease prevalence in juvenile individuals ^24, 25^.

**Table 1.** Green sea turtle samples used in this study. Patient ID, carapace length, weight, origin, condition on arrival, FP tumour score, normal and tumour tissue sources for green sea turtles diagnosed with fibropapillomatosis included in this study. All turtles were patients at the Whitney Laboratory for Marine Bioscience and Sea Turtle Hospital, University of Florida, St Augustine, Florida. Values were recorded at the time turtles were initially admitted to the hospital. FP scores were evaluated using the Southwest Atlantic classification system ^58^. FL, Florida. FP, fibropapillomatosis. GT, Green turtle.

Patient ID	Straight carapace length (cm)	Weight (kg)	Origin	Condition on arrival	FP index tumour score ^58^	Non-tumour tissue source	Tumour tissue source
GT1	32.2	3.8	Volusia, FL	Thin	185.5	Skin biopsy	Left rear flipper (external tumour)
GT2	32.3	4.4	Volusia, FL	Normal body condition	>86.5	Right kidney	Right inguinal area (external tumour), Neck ventral (external tumour regrowth)
GT3	31.4	4.0	St Johns, FL	Thin	None on arrival	Skin biopsy	Right rear flipper (external tumour)
GT4	43.9	7.15	Volusia, FL	Emaciated	>66.6	Lung	Lung (internal tumour)
GT5	32.5	4.1	Volusia, FL	Thin, edematous	>6.6	Skin biopsy	Left rear flipper (external tumour)
GT6	40.0	7.4	Volusia, FL	Thin	>85.6	Skin biopsy	Right front flipper (external tumour)

FP transmission studies in green turtles and spatial patterns of disease spread are consistent with an infectious aetiology ^26, 27^. Transmission to naive captive-reared green turtles via cell-free extracts has also been reported, supporting the possibility of a viral infectious agent ^28^. The disease has been linked with herpesvirus infection, specifically chelonid alphaherpesvirus 5 (ChHV5), also known as fibropapilloma-associated turtle herpesvirus (FPTHV) ^29^. However, a causal relationship between ChHV5 inoculation and disease has not yet been confirmed; ChHV5 infection is also reported in disease-free *C. mydas* populations ^30^ and a tumour-specific ChHV5 viral variant has not yet been identified. Higher disease incidence in turtles exposed to environmental pollutants has been reported ^31, 32^; however, this observation might be explained by other features of near-shore and inshore environments, such as increased population densities ^32– 34^. Transmission of FP via marine leeches ( *Ozobranchus* spp.) and reef cleaner fish has previously been suggested ^35– 37^. Such vector organisms could provide plausible physiological routes for cancer cell transmission ^38^. Spontaneous regression of FP, an observation rarely made in human cancers, has also been described, similar to reports of spontaneous regression in transmissible cancers ^39– 41^.

Peripheral nerve sheath tumours (PNST), including neurofibromas and schwannomas, are the most commonly observed tumours in goldfish ( *Carassius auratus*) ^42, 43^. Schwannoma presents as soft, frequently hemorrhagic, nodules on the skin and subcutaneous tissue ( Figure 1). Tumours express S-100 protein and calretinin, and are proposed to be of Schwann cell ^44^ or fibrocyte ^45^ origin. Although the aetiology of goldfish schwannoma is unknown, a viral aetiology has been hypothesized for the damselfish schwannoma, in which experimentally transplanted tumour cells are capable of causing new growths in naive fish ^46^. A relatively high incidence of schwannoma has been reported in isolated goldfish colonies, suggesting that this tumour may have an infectious origin, and previous reports have hypothesized that this cancer might be transmissible ^42, 43, 47^. Interestingly, both transmissible cancers known in Tasmanian devils arose from the Schwann cell lineage ^48, 49^. It is possible that goldfish schwannoma may represent a transmissible clonal cancer wherein social behaviour during spawning could provide a mechanism of cancer cell transfer. Moreover, growing evidence suggests that aquatic environments may provide favourable conditions for transfer of genetic material, even transmissible cancer cells ^50– 53^.

Here, we analysed microsatellite repeat loci in host and tumour tissue from diseased *C. mydas* and *C. auratus* in order to determine whether either FP or goldfish schwannoma had clonal origins. We found that in both tumour types, genotypes of neoplastic cells matched those of their hosts, strongly arguing that cancer cells are host-derived and excluding clonal transmission of the analysed tumours.

## Methods

The animal studies described below adhered to the Animal Research: Reporting of *In Vivo* Experiments (ARRIVE) guidelines ^54– 56^. All efforts were made to minimize the animals' suffering and to reduce the number of animals used for experiments.

### Green Sea Turtle Fibropapillomatosis

***Ethics***. Tissue sampling was carried out under permit number MTP-18-236 from the Florida Fish and Wildlife Conservation Commission (FWC) and with ethical approval from the University of Florida Institutional Animal Care and Use Committee (IACUC), under protocol number 201909289. No additional stress or suffering was experienced by the sea turtle patients in relation to this sampling, and the research sampling in no way interfered with the veterinary care and rehabilitation of these wild animals. All sampling was at the discretion of the attending veterinarian, and samples were obtained during necropsy or during rehabilitation-related tumour removal surgeries during which patients were anesthetized.

***Sample collection and diagnosis***. Tissues from six juvenile green sea turtles were collected in 2017 and 2018 at the Whitney Laboratory for Marine Bioscience and Sea Turtle Hospital, St Augustine, Florida, as previously described ^57^. The sex of juvenile individuals is not readily determinable.

Turtles were held in 240 cm diameter circular tanks, holding 2,270 litres of continuously filtered sea water. The researchers had no role in the husbandry or housing of the turtles, these are not experimental animals, rather endangered animals undergoing rehabilitative care with the ultimate goal of their release back to the ocean. Sample collection was opportunistic, without explicit sampling design. The only inclusion criteria applied was that only stranded sea turtles afflicted by external FP tumour growth were eligible for the study. As a patient-matched (tumour and non-tumour samples) analysis approach was employed six turtles were deemed to be an appropriate number to confirm whether tumour genotypes matched that of the host animal.

Internal tumour and host tissue samples were obtained during routine necropsy of animals euthanised due to inoperable internal tumour burdens (as per governing FWC rehabilitation guidance). Researchers involved in the study played no role in euthanasia decisions. Euthanasia was performed on a case-by-case basis at the discretion of the attending veterinarian with express permission of FWC and in line with disease severity, quality of life and likely rehabilitation outcome considerations, as per the governing sea turtle rehabilitation-related FWC guidance. External fibropapilloma tumours were surgically removed by laser resection as part of routine rehabilitative care.

Tumour and host tissue biopsies ( Table 1) were stored at -80°C until processing. Gross and histopathological examinations were performed by veterinary pathologists to confirm FP diagnosis. Non-tumour biopsy sites were identified by gross examination by the attending veterinarian; such regions were confirmed visually to be tumour-free and not bordered by any tumour regions by attending veterinary technicians and researchers, as previously described ^57^.

For each individual, FP severity was assessed according to the Southwest Atlantic score system ^58^ ( Table 1). The turtles included in this study had a tumour score range of mild to severe (>6.6 to 185.5). However, individual GT3 was not scored upon admittance to the hospital ( Table 1).

***DNA extraction***. Representative tissue sampled from tumour and host biopsies was used for DNA extraction using the Qiagen DNeasy Blood and Tissue Kit (Qiagen, Hilden, Germany) according to manufacturer’s instructions. DNA was quantified using a Thermo Scientific NanoDrop 2000 UV-Vis Spectrophotometer (Thermo Fisher Scientific, USA).

***Polymerase chain reaction (PCR)***. Polymorphic microsatellite loci (A6, B103, B123, C102, D108) were amplified using primers designed by Dutton and Frey ^59^ and modified by the addition of a 19 bp M13 tag (AGGAAACAGCTATGACCAT) to the 5’ end of each forward primer. PCR was performed using an Eppendorf 6331 Nexus Gradient MasterCycler Thermal Cycler (Eppendorf, Hamburg, Germany) with conditions as follows: 2 µl of genomic DNA was amplified in a total volume of 20 μl containing 0.6 μM of each primer, 0.2 mM of each deoxynucleotide triphosphate (dNTP), 1.5 mM of MgCl _2_, 2 μl of 10x ThermoPol Reaction Buffer (New England Biolabs, Ipswich, United States), and 0.2 μl of Taq DNA polymerase (New England Biolabs, Ipswich, United States) per reaction. Cycling conditions were 94°C for 2 min; followed by 5 cycles of 94°C for 20 s, 57°C for 20 s, 72°C for 20 s (with a decrease by 1°C, to annealing temperature, each cycle); followed by 30 cycles of 94°C for 20 s, 50°C for 20 s, 72°C for 20 s; followed by addition of 1 μl of a 10 μM 5’-FAM-labelled M13 oligo followed by 7 cycles of 94°C for 20 s, 48°C for 20 s, 72°C for 20 s; and a final extension step at 72°C for 10 min. PCR products were submitted for fragment length analysis at the University of Florida’s Gene expression and Genotyping Core Facility (University of Florida, Gainesville, Florida, USA) using a 3730 DNA Analyzer with GeneScan 600 LIZ Size Standard v2.0 (Applied Biosystems, Waltham, Massachusetts, USA).

***Microsatellite analysis***. Allele determinations were made by analysing electropherograms using GeneMarker software (v2.6.3, SoftGenetics, State College, Pennsylvania).

### Goldfish Schwannoma

***Ethics***. Permission to perform experiments on goldfish was granted by the Italian Ministry Center for Scientific and Ethics Committee, under permit DM 39.03.05". Experiments were carried out at the Sicilian Centre for Experimental Fish Pathology (CISS), Establishment for Users recognized by the Italian Ministry of Health, according to decree no. 39 of 19/03/2005.

***Sample collection, diagnosis and DNA extraction***. Goldfish originated from a commercial source in Palermo, Italy. The first tumour was recorded 3 years before sample collection and in the intervening time tumours became recognizable in eleven other fish. Twelve PNST-affected adult goldfish (ten female, two male) were collected from the same tank containing only one unaffected goldfish. Sample collection was opportunistic, without explicit sampling design. The fish were moved to the Sicilian Centre for Experimental Fish Pathology, where the experiments described below were performed. Healthy goldfish were obtained from either a commercial source in Messina, Italy or bred at the CISS, an establishment authorized for production of fish for experiments.

After an anaesthesic bath of 100 mg/l tricaine methanesulfonate lasting 3-4 minutes, incisional tumour biopsies were taken from four of the female PNST-affected goldfish. Tumour diagnosis was confirmed by histopathology and immunohistochemistry. A matched blood sample was taken from the caudal vein to obtain host tissue for genetic comparison. Tumoural tissues were microdissected and DNA was extracted from each tumour and blood sample using the Qiagen DNeasy Blood and Tissue Kit (Qiagen, Hilden, Germany) according to manufacturer’s instructions, for analysis and comparison of microsatellite genotypes.

***PCR***. Polymorphic microsatellite loci (GF1, GF17, Ca07, Ca08) were amplified using primers described by Zheng *et al*. ^60^ and Yue *et al*. ^61^. PCR was performed with conditions as follows: 20 ng of genomic DNA was amplified in a total volume of 20 μl containing 0.6 μM of each primer, 0.2 mM of each dNTP and 0.2 units of Taq DNA polymerase (Qiagen, Hilden, Germany) per reaction. PCR was performed using cycling conditions described in Pye *et al*. ^13^.

***Microsatellite analysis***. Microsatellite allele lengths were identified by fragment analysis using an ABI 3730 DNA analyzer (Applied Biosystems, Foster City, California), and analysed using GeneMarker software (Softgenetics Inc, State College, Pennsylvania).

***Experimental transmission via water exposure***. Five PNST-affected goldfish (three female, two male) were reared in the same tank with five healthy goldfish (two female, three male). Tank dimensions were approximately 100.5 cm long × 30.7 cm wide × 47 cm deep. Social manifestations were allowed, including spawning behaviour. Fish were observed at weekly intervals for the presence of externally visible lesions. After one year, seven fish were euthanized and necropsies performed. The three female PNST-affected goldfish were not euthanized and used in a further experiment (Experimental transmission via scarification). Euthanasia was performed by an overdose of anesthetics (tricaine methanesulfonate). This was necessary for complete pathological examination.

***Experimental transmission via scarification***. Five female PNST-affected goldfish and five female healthy goldfish were anaesthetized with 120 mg ml ^-1^ MS 222 at pH 8. Using scalpels, 50 mm ^2^ of skin was scarified in each of the healthy group fish and about 50mm ^3^ incisional biopsies were taken from the tumours in the PNST-affected group. Biopsies were rubbed on the scarified areas. Fish were then placed in fresh water and observed at weekly intervals for the presence of externally visible lesions. After one year, all fish were euthanized and necropsies performed. Euthanasia was performed as described above and was necessary for complete pathological examination.

***Experimental transmission via inoculation***. Five female PNST-affected goldfish and five female healthy goldfish were anaesthetized as described above and submitted to another experimental transmission of tumoural cells. Using a 20 G needle connected to a 2.5 ml syringe, tumours were gently aspirated in order to collect a narrow cylinder of live tumoural tissue, which was quickly inoculated under the dorsal fin skin of healthy fish. Fish were then placed in fresh water and observed at weekly intervals for the presence of externally visible lesions. After one year, all fish were euthanized and necropsies performed. Euthanasia was performed as described above and was necessary for complete pathological examination.

## Results

We analysed matched tumour and host samples from six FP-affected green sea turtles at five polymorphic microsatellite loci. In total, 24 alleles were identified in the population ( Table 2) ^56, 62^. At all loci, FP tumour genotypes were identical to matched host genotypes ( Table 2).

**Table 2.** Microsatellite analysis of green sea turtle fibropapillomatosis. Microsatellite genotypes at five polymorphic microsatellite loci (A6, B103, B123, C102, D108) across six individuals (GT1-GT6). The lengths of the alleles found at each locus in matched tumour and host tissues are indicated. An M13F tag sequence (5′-AGGAAACAGCTATGACCAT-3′) was added to the 5′ end of each forward primer; this 19 bp sequence is included in the size of the allele. Tumours (T) and matched normal individuals (N) share identical alleles across all loci.

		GT1		GT2		GT3		GT4		GT5		GT6	
		N	T	N	T	N	T	N	T	N	T	N	T
**Microsatellite**	A6	142, 142	142, 142	138, 142	138, 142	142, 142	142, 142	136, 138	136, 138	136, 142	136, 142	138, 142	138, 142
	B103	177, 180	177, 180	171, 179	171, 179	171, 180	171, 180	180, 180	180, 180	180, 183	180, 183	171, 180	171, 180
	B123	235, 235	235, 235	234, 234	234, 234	234, 234	234, 234	234, 234	234, 234	234, 240	234, 240	235, 235	235, 235
	C102	252, 252	252, 252	252, 262	252, 262	252, 264	252, 264	252, 262	252, 262	256, 262	256, 262	252, 252	252, 252
	D108	272, 288	272, 288	268, 275	268, 275	271, 271	271, 271	267, 271	267, 271	243, 283	243, 283	272, 279	272, 279

In *C. auratus,* we analysed the genotypes of four matched tumour and host samples across four polymorphic microsatellite loci. Overall, 16 alleles were identified ( Table 3). At all loci, tumour and matched host tissues shared identical genotypes ( Table 3). Transmission of PNST from affected to naive goldfish through water exposure was not observed during laboratory proximity experiments. Moreover, inoculation of healthy goldfish with schwannoma cells by rubbing scarified skin with tumour biopsies, or by subcutaneous implantation of tumour biopsies, did not result in engraftment. Furthermore, no post-challenge complications were recorded.

**Table 3.** Microsatellite allele analysis of goldfish schwannoma. Microsatellite genotypes at four polymorphic microsatellite loci (GF1, GF17, Ca07, Ca08) in four individuals (G1-G4). The lengths of the allele(s) found at each locus in matched tumour and host tissues are indicated. An M13F tag sequence (5′-AGGAAACAGCTATGACCAT-3′) was added to the 5′ end of each forward primer; this 19 bp sequence is included in the size of the allele. Tumours (T) and matched hosts (N) share identical alleles across all loci.

		G1		G2		G3		G4	
		N	T	N	T	N	T	N	T
**Microsatellite**	GF1	330, 330	330, 330	330, 330	330, 330	316, 316	316,316	327,327	327,327
	GF17	204, 204	204, 204	184, 184	184, 184	184, 184	184, 184	215,215	215,215
	Ca07	142, 146	142, 146	161, 161	161, 161	145, 145	145,145	137,137	137,137
	Ca08	201, 213	201, 213	192, 192	192, 192	192, 202	192, 202	202,247	202,247

## Discussion

Microsatellite genotyping confirmed that the examined fibropapillomatosis tumours in green sea turtles are of host origin, and indicated that these tumours were not clonally transmitted between animals. Investigating the interaction of viral and environmental cofactors, such as water temperature, ultraviolet (UV) radiation and marine toxin exposure, that may lead to FP pathogenesis will be an interesting area for future study and may provide valuable information about how host genetics, host immunity, and ecological environments influence cancer growth and how disease spreads through marine environments ^57, 63^. ChHV5 has been detected in the tank water of FP-afflicted green sea turtles, and FP-afflicted turtles exhibiting weaker immune activation had worse clinical outcomes ^37, 63^. Recently, green sea turtle papillomavirus 1 (CmPV1) was reported in 47% of FP tumours analysed, suggesting the potential of multiple viruses as cofactors in FP disease ^64^. It is also interesting to note that FP lesions, along with concurrent ChHV5 infection, have been reported in the eastern box turtle ( *Terrapene carolina*), a terrestrial turtle species ^65^.

Analysis of polymorphic microsatellite loci showed that goldfish schwannoma tumour genotypes consistently match corresponding host genotypes, and implies that these tumours did not derive from a single clonal origin. Results of the cohabitation and inoculation experiments indicated that these tumours were not readily transmitted by contact with water from affected goldfish, or by implantation of tumour cells, and suggest that this disease may not have an infectious aetiology. Instead, genetic susceptibility, perhaps influenced by reduced genetic diversity may contribute to disease in domestic goldfish ^66^.

While we did not find evidence of transmissible cancer, the genotyping and experimental transmission studies described here are limited to a small set of samples and cannot exclude the existence of different tumour subtypes in these species, some of which may be transmissible. Indeed, the co-occurrence of transmissible and non-transmissible forms of bivalve haemic neoplasia in mussels confirms that larger-scale sampling and genetic identification may be required in order to definitively rule out direct cancer cell transmission ^17, 67^. Furthermore, although care was taken to biopsy neoplastic sites in both cancers, it is worth noting that we were unable to confirm the proportion of neoplastic cells in the samples analysed in this study.

Although transmissible cancer clones are thought to emerge rarely, their numbers and distributions in wildlife populations are difficult to assess ^68, 69^. The current work, together with a previous study of urogenital carcinoma in California sea lions, argues against transmissible cancer aetiologies for three well-recognised animal cancers ^8^.

Testing the hypothesis of transmissible cancer is an important step in understanding pathological processes involved in animal cancers and provides new research opportunities for animal disease biomonitoring and control ^2^. Like pathogens and parasites, cancer, especially transmissible cancer, can have a negative impact on host fitness in wildlife populations and may be an important, but often overlooked, feature of animal ecosystems ^70^. Future analysis of goldfish schwannoma and green turtle fibropapillomatosis will further reveal the mechanisms of these diseases and improve our understanding of how cancer occurs in animals in aquatic environments.

## Data availability

### Underlying data

Dryad: Genotype data not consistent with clonal transmission of sea turtle fibropapillomatosis or goldfish schwannoma. https://doi.org/10.5061/dryad.1zcrjdfsg ^62^.

This project contains the following underlying data:

- Fragment analysis data files

Data are available under the terms of the Creative Commons Zero "No rights reserved" data waiver (CC0 1.0 Public domain dedication).

Zenodo: Genotype data not consistent with clonal transmission of sea turtle fibropapillomatosis or goldfish schwannoma. https://doi.org/10.5281/zenodo.5172413 ^56^.

This project contains the following underlying data:

- Green_sea_turtle_FP_microsat_annotated_gel_images.pdf

Data are available under the terms of the Creative Commons Attribution 4.0 International license (CC-BY 4.0).

### Reporting guidelines

Zenodo: ARRIVE Essential 10 checklists for ‘Genotype data not consistent with clonal transmission of sea turtle fibropapillomatosis or goldfish schwannoma’ https://doi.org/10.5281/zenodo.5172413 ^56^.

Data are available under the terms of the Creative Commons Attribution 4.0 International license (CC-BY 4.0).
