# Supplementary material for: Genotype data not consistent with clonal transmission of sea turtle fibropapillomatosis or goldfish schwannoma
Source: Wellcome Open Res. 2021 Sep 2;6:219. [Version 1] doi: 10.12688/wellcomeopenres.17073.1 (PMC8459624; doi:10.12688/wellcomeopenres.17073.1)
Supplement: Peer review history

# Peer review: Reviewer response for version 1

**DOI:** 10.21956/wellcomeopenres.18860.r45838

**Published:** 2021-09-22

**Authors:** Ariberto Fassati

**Affiliations:** 1 Division of Infection & Immunity, Institute of Immunity and Transplantation, University College London, London, UK

In this paper, the authors examined the possible clonal origin of two tumours of unknown etiology and suspected transmissibility in the sea turtle *Chelonia mydas* and the gold fish. The authors obtained DNA from several affected individuals (tumour biopsies and control normal tissue) and carried out DNA microsatellite polymorphisms analysis. The results showed that, in every case examined, the tumour matched the host and therefore the clonal origin of these cancers was excluded.

 The paper is well written, the rationale clearly explained and even if the results are negative, it will be useful to other colleagues to know about these findings. Actually, I would welcome a systematic analysis of other potential candidates.

 The manuscript may be improved by providing some sense of how diverse microsatellite polymorphisms are in these species, if known, and more info in the Results section on why these particular regions were selected. Lastly, based on the microsatellite polymorphisms diversity, can a p value be calculated for the likelihood that these tumours are not clonal?

Is the work clearly and accurately presented and does it cite the current literature?

Yes

If applicable, is the statistical analysis and its interpretation appropriate?

I cannot comment. A qualified statistician is required.

Are all the source data underlying the results available to ensure full reproducibility?

Yes

Is the study design appropriate and is the work technically sound?

Yes

Are the conclusions drawn adequately supported by the results?

Yes

Are sufficient details of methods and analysis provided to allow replication by others?

Yes

Reviewer Expertise:

Transmissible cancers, HIV-1

I confirm that I have read this submission and believe that I have an appropriate level of expertise to confirm that it is of an acceptable scientific standard.

# Peer review: Reviewer response for version 1

**DOI:** 10.21956/wellcomeopenres.18860.r45745

**Published:** 2021-09-22

**Authors:** Michael Metzger

**Affiliations:** 1 Pacific Northwest Research Institute, Seattle, WA, USA

The Ní Leathlobhair paper investigates the hypothesis that two animal cancers (in green sea turtles and goldfish) could be transmissible cancers, like those found in dogs, Tasmanian devils, and bivalves. They clearly articulate the reason that this hypothesis was suggested in each case (primarily that there is evidence that both diseases occur in outbreaks and likely have an infectious etiology). The authors used microsatellites and convincingly showed that it is highly unlikely that these cancers are transmissible.

 There are two main technical reasons that these studies might be a false negative result (ie. that the cancer could still possibly be transmissible). The first is that the cancer sample could have an insufficient amount of cancer cells. If the cancer sample was only partially cancer cells (<50%) then it can be difficult to detect the alleles corresponding to the cancer. The second is that the primers might not amplify the corresponding alleles in the cancer genome, due to mutations in the primer sequence. It is less likely that this would occur at multiple loci, so the use of multiple loci makes this less of a concern. This could also occur in the case of a cross-species transmissible cancer, but that is currently believed to be rare. The authors address the limitations of the study appropriately.

 We know from the finding of transmissible cancers in many cases of disseminated neoplasia that there remain many transmissible cancer lineages that have yet to be found within marine bivalves, but it is unclear if there are other unknown transmissible cancers in other species, particularly those in the marine environment. Despite some speculation, there is no real way to determine whether there are unknown transmissible cancers in different species without experimental work like this study. It is an important addition to the field.

**Minor comments:**

1. I see the fragment analysis data for the sea turtle, but I did not find the goldfish schwannoma cell fragment analysis data available. Given the likelihood that cancer samples reflect a mix of cancer and host tissue, it is important to be able to observe the peaks to determine whether there are any low peaks which might correspond to a small fraction of transmissible cancer in the sample.
2. Additionally, providing a figure which shows an example of the fragment analysis trace files from cancer and host, showing the absence of cancer-specific alleles could be helpful. Analyzing the fragment analysis files is non-trivial, so the display of an example in an image format could be very useful to reassure readers that you are not missing low peaks from rare cancer cells.

Is the work clearly and accurately presented and does it cite the current literature?

Yes

If applicable, is the statistical analysis and its interpretation appropriate?

Not applicable

Are all the source data underlying the results available to ensure full reproducibility?

Partly

Is the study design appropriate and is the work technically sound?

Yes

Are the conclusions drawn adequately supported by the results?

Yes

Are sufficient details of methods and analysis provided to allow replication by others?

Yes

Reviewer Expertise:

Transmissible Cancer, retroelements

I confirm that I have read this submission and believe that I have an appropriate level of expertise to confirm that it is of an acceptable scientific standard.

# Peer review: Reviewer response for version 1

**DOI:** 10.21956/wellcomeopenres.18860.r45744

**Published:** 2021-09-20

**Authors:** Antoine M. Dujon

**Affiliations:** 1 Centre for Integrative Ecology, School of Life and Environmental Sciences, Deakin University, Geelong, Vic, Australia; 2 CANECEV-Centre de Recherches Ecologiques et Evolutives sur le cancer (CREEC), Montpellier, France

The article by Ní Leathlobhair *et al.* investigates if two neoplastic diseases (the fibropapillomatosis in sea turtles and the schwannoma in goldfish) are transmissible cancers (clonal neoplastic cells that evolved the ability of infect multiple individuals). The authors used microsatellites to genotype both the host and the tumours under the assumption that a transmissible cancer would have a different genotype than the host. They found that for both species the tumours samples and the host samples had the same genotype and concluded the samples they analysed were not transmissible cancers. In addition the authors performed transmission experiments on goldfishes to investigate if the tumours were transmissible under laboratory conditions. They found no evidence of such transmission.

 You will find bellow my comments and suggestions.

**Introduction:**

 I think the introduction would strongly benefit from a paragraph detailing why it is important to improve our ability to detect transmissible cancers in wildlife species. Even in the case of transmissible cancers being rare, they can have strong effects on the host populations. For me it means the results presented in this study are of interest, because while tumours in sea turtles and goldfishes can have strong negative effects on the host, those effects could be worse if they were transmissible cancers. 

 Here is a list of arguments that could figure in that paragraph (or alternatively in a paragraph in the discussion).

- Transmissible cancer can cause mass mortalities in the host populations as evidenced in Tasmanian devils (>80% of the devils wiped out in >20 years) and marine bivalve populations (see Mateo *et al.* 2016 ^1^ and references herein for bivalves).
- Tumours, in general, have the potential to reshape biotic interactions (as evidenced in hydras ^2^) and more broadly to modify the host behaviour ^3^.
- Transmissible cancers can lead to trophic cascades and other important changes in ecosystems (e.g. proliferation of invasive species), which are especially well documented in Tasmanian devils ^4^^,^^5^^,^^6^.
- Transmissible cancers are likely more common than we thought which is mentioned in the manuscript. I suggest to read the following paper, Dujon *et al.* (2020) ^7^) in which I provide a first quantitative estimation of the potential number of transmissible cancers in mammal and bivalve species.
- We don’t know very well how environmental changes, such as climate change and other environmental degradations, may affect transmissible cancers ^8^^,^^9^. It this therefore important to improve our ability to detect them to establish baseline data.

The following paper is cited in the introduction “Land Use, Macroalgae, and a Tumor-Forming Disease in Marine Turtles”. I recommend to read the following paper, Work *et al.* (2014) ^10^ before deciding to cite this study since it is considered to be a controversial study.

**Material and methods:**

 The choice of fibropapillomatosis (FP) as a potential candidate for being a transmissible cancer seems a bit odd, since so far, a significant body of literature is pointing toward a viral cause. The main issue with ChHV5, is it is difficult to satisfy the Koch postulate because the virus is hard to cultivate in the lab. It is very likely the virus is initiating the tumours; but we are still unclear what are the environmental condition acting as promotor (reviewed in Jones *et al.* 2016 ^11^). As such I was not expecting the authors to find that FP was a transmissible cancer, which they confirmed with their analyses.

 I am not fully convinced microsatellites alone are sufficient to be able to differentiate transmissible cancers from the host (and that the risk of false negatives is not greatly increased). For example, Hammel *et al.* (2021) ^12^ relied on a large number of SNPs to distinguish between transmissible cancer and the host in mussels. I think the authors must justify of their choice of using microsatellites and why they think the method is powerful enough to detect transmissible cancers.

**Discussion:**

 A limitation of the study (mentioned by the authors and which they could develop a bit more) is that for sea turtles a small number of tumours samples were obtained from only two study sites while FP has now reached a panzoonotic status. Similarly, a small number of samples were obtained from goldfishes. In the following paper, Bramwell *et al.* (2021) ^8^ sensitivity analyses were performed to investigate, given a transmissible cancer prevalence, how many individuals you would need to sample to be able to detect it with a probability of 0.95 or 0.99. For a low prevalence 100’s of individuals are often required (assuming the method to detect the transmissible cancer works perfectly).

 The transmission experiments were also performed on a small number of individuals, but I can see the worth of those experiments as preliminary results.

 For the fourth paragraph of the discussion see the following paper, Dujon *et al.* (2021) ^9^ I mentioned before since it provides a quantitative estimation of the number of transmissible cancers in bivalves and mammals.

 In conclusion I think we need more studies systematically investigating if cancers observed in wildlife are transmissible or not (alike Ní Leathlobhair *et al.* study, but with an adequate sample size). Even if they return “negative” results, such studies allow to establish a baseline which we are currently lacking.

**References:**

 Species name in references 24, 27, 36, 60 are not in italics. Worth double checking the references.

Is the work clearly and accurately presented and does it cite the current literature?

Partly

If applicable, is the statistical analysis and its interpretation appropriate?

Not applicable

Are all the source data underlying the results available to ensure full reproducibility?

Yes

Is the study design appropriate and is the work technically sound?

Yes

Are the conclusions drawn adequately supported by the results?

Yes

Are sufficient details of methods and analysis provided to allow replication by others?

Yes

Reviewer Expertise:

Marine Biology, Quantitative Ecology, Ecology and Evolution of Cancer

I confirm that I have read this submission and believe that I have an appropriate level of expertise to confirm that it is of an acceptable scientific standard, however I have significant reservations, as outlined above.

## References

[1] : Field and laboratory transmission studies of haemic neoplasia in the soft-shell clam, Mya arenaria, from Atlantic Canada.*J Fish Dis*.2016;39(8) : 10.1111/jfd.12426913-2710.1111/jfd.1242626687447

[2] : Tumors (re)shape biotic interactions within ecosystems: Experimental evidence from the freshwater cnidarian Hydra.*Sci Total Environ*.2021;803: 10.1016/j.scitotenv.2021.14992314992310.1016/j.scitotenv.2021.14992334487898

[3] : Animal behaviour and cancer. *Animal Behaviour*.2015;101: 10.1016/j.anbehav.2014.12.00119-2610.1016/j.anbehav.2014.12.001

[4] : Disease-induced decline of an apex predator drives invasive dominated states and threatens biodiversity.*Ecology*.2016;97(2) : 10.1890/15-0204.1394-40510.1890/15-0204.127145614

[5] : Top carnivore decline has cascading effects on scavengers and carrion persistence. *Proceedings of the Royal Society B: Biological Sciences*.2018;285(1892) : 10.1098/rspb.2018.158210.1098/rspb.2018.1582PMC628394730487308

[6] : Trophic cascades following the disease-induced decline of an apex predator, the Tasmanian devil.*Conserv Biol*.2014;28(1) : 10.1111/cobi.1215263-7510.1111/cobi.1215224024987

[7] : Transmissible cancers in mammals and bivalves: How many examples are there?: Predictions indicate widespread occurrence.*Bioessays*.2021;43(3) : 10.1002/bies.202000222e200022210.1002/bies.20200022233210313

[8] : A review of the potential effects of climate change on disseminated neoplasia with an emphasis on efficient detection in marine bivalve populations. *Science of The Total Environment*.2021;775: 10.1016/j.scitotenv.2021.14513410.1016/j.scitotenv.2021.145134

[9] : Transmissible cancers in mammals and bivalves: How many examples are there?: Predictions indicate widespread occurrence.*Bioessays*.2021;43(3) : 10.1002/bies.202000222e200022210.1002/bies.20200022233210313

[10] : The story of invasive algae, arginine, and turtle tumors does not make sense.2014; 10.7287/peerj.preprints.53910.7287/peerj.preprints.539

[11] : A review of fibropapillomatosis in Green turtles (Chelonia mydas).*Vet J*.2016;212: 10.1016/j.tvjl.2015.10.04148-5710.1016/j.tvjl.2015.10.04127256025

[12] : Prevalence and polymorphism of a mussel transmissible cancer in Europe.*Mol Ecol*.2021; 10.1111/mec.1605210.1111/mec.1605234192383PMC8716645
